# Nanolayered Structures and Nanohybrids Based on a Ternary System Co/Ti/Zn for Production of Photo-Active Nanocomposites and Purification of Water Using Light

**DOI:** 10.3390/nano14010093

**Published:** 2023-12-28

**Authors:** Osama Saber, Aya Osama, Nagih M. Shaalan, Mostafa Osama

**Affiliations:** 1 Department of Physics, College of Science, King Faisal University, P.O. Box 400, Al-Ahsa 31982, Saudi Arabia; ayamohamed19984@yahoo.com (A.O.); nmohammed@kfu.edu.sa (N.M.S.); mostafa.osama8664@gmail.com (M.O.); 2 Egyptian Petroleum Research Institute, Nasr City, P.O. Box 11727, Cairo 11765, Egypt; 3 Physics Department, Faculty of Science, Assiut University, Assiut 71516, Egypt

**Keywords:** Co/Ti/Zn nanocomposites inorganic/organic nanohybrids, cobalt/titanium/zinc nanolayers, low band gap energy, water purification, photo catalytic degradation

## Abstract

Water pollution has emerged as a major challenge for the scientific community because of the rapid expansion of the population and the industrial sector in the world. The current study focuses on introducing a new track for designing new optical nanocomposites for purifying water in addition to providing a new additive for building new nanohybrids. These targets were achieved through building a ternary system of Co/Ti/Zn nanocomposites and nanolayered structures. The Co/Ti/Zn nanolayered structures were prepared and intercalated by different kinds of organic acids: monocarboxylic and dicarboxylic acids. Long chains of organic acids were used to construct series of organic–inorganic nanohybrids. X-ray diffraction, thermal analyses, Fourier Transform Infrared spectroscopy, and scanning electron microscopy confirmed the formation of nanolayered structures and nanohybrids. The optical properties of the nanolayered structure showed that the Co/Ti/Zn LDH became photo-active compared with the usual Al/Zn LDH because of the reduction in the band gap energy from 5.3 eV to 3.3 eV. After thermal treatment, a highly photo-active nanocomposite was produced through observing more reduction for the band gap energy to become 2.8 eV. In addition, the dye of Acid Green 1 completely decomposed and converted to water and carbon dioxide during 17 min of UV radiation by the dual Co/Ti-doped zinc oxide nanocomposite. In addition, the kinetic study confirmed that the high optical activity of the dual Co/Ti-doped zinc oxide nanocomposite accelerated the degradation of the green dyes. Finally, from these results it could be concluded that designing effective nanocomposite for purification of water was accomplished through converting 2D nanolayered structures to a 3D porous structure of Ni/Ti/Zn nanocomposites. In addition, a new additive was achieved for heterostructured hybrids through building new Co/Ti/Zn/organic nanohybrids.

## 1. Introduction

The rapid expansion of the industrial sector in the world leads to different kinds of environmental problems. The main problems are related to water resources because industrial and human activities release various pollutants toward water media. The organic and colored pollutants are concentrated and accumulated inside soil and plants, becoming more dangerous for the ecosystem and human health. The usual techniques, which are used for solving these problems, have many disadvantages. In addition to producing secondary pollutants, they are not effective for all pollutants. Therefore, the techniques based on using light to convert the organic pollutants to water and carbon dioxide are favorable for solving the environmental problems of water. Photocatalytic degradation is one of the most familiar processes belonging to these techniques. Many researchers used photocatalytic reactions for removing pollutants [1,2,3,4,5,6,7]. In these studies, the researchers tried to prepare and design effective optical materials because the performance of the photocatalytic processes depends mainly on the structure of the optical materials. A wide range of structures were used as photocatalysts for purifying water from industrial pollutants [8,9].

Among the different kinds of materials, nanolayered structures based on layered double hydroxides (LDHs) have many advantages for preparing optical active materials. LDHs consist of several nanolayers connecting through pillars with a large interlayered space [3,4,5]. These nanolayers have a cationic nature because they contain different metals with different valences such as di-, tri-, and tetra-valent metals. The presence of the different valences creates a cationic nature for the nanolayers [3,8,9]. The pillars have an anionic nature to neutralize the cationic nature of the nanolayers. Depending on the nature and the size of the anionic pillars, the internal spacing among the nanolayers can be controlled and changed to appear as a large spectrum of known or unknown properties. By using organic pillars, different kinds of organic–inorganic nanohybrids can be formed to produce an almost unlimited set of new compounds [3,4,5].

Xu et al. [10,11,12] indicated that the Zn-containing LDHs and their derivatives such as Zn-Al LDH and Zn-Ti LDH are active materials for photocatalytic activities. In addition, Parida et al. [13,14,15] reported that the high efficiency of photocatalysts for removing different kinds of industrial pollutants depends on conduction band edges, valence band edges, and band gap energy. The results of Yuan et al. [16,17,18] showed that the photocatalytic activity of the surfaces of LDHs is effective because of the high positive valence band position of the LDH (+3.620 V). Li et al. [19] indicated that the conduction band (CB, −0.61 V) of Zn-Ti LDH and its valence band (VB, +2.52 V) led to a low band gap energy of ~3.13 eV and the formation of hydroxyl radicals, which were effective for photocatalytic reactions. In order to modify Zn-LDHs materials, many scientists used thermal treatment, intercalation reactions, and surface decoration for developing photocatalysts to be effective for photocatalytic reactions [20,21,22,23,24]. Recently, many reports were issued on LDHs structures [25,26,27,28,29]. Most of them concentrated on applications and synthesis of LDHs to study the removal of pollutants through adsorption technique or the photocatalytic degradation processes. However, no data or scientific papers were published on designing effective photocatalysts based on the ternary system of cobalt/titanium/zinc LDHs and its nanohybrids for the photocatalytic degradation of green dyes.

In 2023, many researchers [30,31,32,33,34,35,36,37,38,39,40] employed nanocomposites and nanohybrids to be used as effective photocatalysts and harnessing light radiation for breaking down different kinds of dyes in contaminated wastewater such as Malachite Green, Rhodamine B, Methyl Orange, and Congo red in addition to food and juices dyes. In these processes, the highly reactive hydroxyl (^•^OH) radicals are generated to convert water pollutants to benign end products, such as carbon dioxide and water.

In this trend, the current study focused on designing effective optical materials for removing and converting green dyes to carbon dioxide and water in a few minutes using UV light. Through the nanolayered structures based on the ternary system of metals (Co, Ti, and Zn), the regular arrangements of the elements inside the nanolayers were used for designing photo-active nanocomposites in addition to heterostructured nanohybrids. The optical active nanocomposites were used as a driving force for developing photocatalytic technology to become more favorable and a good solution for the environmental problems. Therefore, the photo activity of the prepared nanolayered structures and nanocomposites were measured and compared through photocatalytic degradation of the green dyes. In addition, the acceleration of the photo catalytic reactions was studied by kinetic studies.

## 2. Materials and Methods

### 2.1. Preparation of Nanolayered Structures

The ternary system of Co/Zn/Ti LDH was synthesized through urea hydrolysis. By urea hydrolysis at 80 °C, carbonate and cyanate anions were released in the reaction to be used as pillars for building the nanolayered structures of LDHs. At the same time, the medium of the reaction was transformed from an acidic character to an alkaline character through releasing ammonia. The alkaline medium was favorable for building and precipitating the nanolayers of LDHs. The ratio of the ternary system Co/Zn/Ti LDH was 1:5:1; respectively. Typically, an aqueous solution of zinc (II) chloride (Zn Cl_2_; 4.95 × 10^−2^ M), cobalt (II) chloride (9.95 × 10^−3^ M), and titanium(IV) chloride (TiCl_4_; 9.95 × 10^−3^ M) was mixed with a urea solution under vigorous stirring and heating. After 12 h, the product was separated by a filtration process. Deionized and distilled water were used for washing several times. The product was kept in the vacuum for 24 h for drying and labeled by Co/Zn/Ti LDH.

For comparison, the conventional Zn/Al LDH was synthesized by the same method. The prepared material Co/Zn/Ti LDH was calcined at 500 °C and 900 °C to produce two nanocomposites Co/Zn/Ti-500 and Co/Zn/Ti-900; respectively. Moreover, the conventional Zn/Al LDH was treated at 500 °C to produce the Al-doped zinc oxide Zn/Al-500.

### 2.2. Preparation of Nanohybrids

The organic monocarboxylic acids sodium salts; n-capric acid (CH_3_(CH_2_)_8_COOH), myristic acid (CH_3_(CH_2_)_12_COOH), and stearic acid (CH_3_(CH_2_)_16_COOH) were obtained from WAKO Company (Tokyo, Japan). The supplies of dicarboxylic acids sodium salts (suberic acid COOH(CH_2_)_6_COOH and sebacic acid COOH(CH_2_)_8_COOH) were from TCI Company (Tokyo, Japan). The nanohybrids were formed through anions exchange reactions. Typically, the appropriate concentration of organic acid (0.2 M) was prepared through dissolving 0.002 mol of the sodium salt of aliphatic acids in 10 mL of deionized–distilled water. By using ultrasonic technique, this aqueous solution of organic acid was mixed with 0.5 g of the prepared Co/Zn/Ti LDH for performing intercalation reactions. This process was achieved under an argon atmosphere with strong stirring at room temperature for 24 h. The product was filtrated and washed many times by deionized–distilled water. The fine powder was obtained after drying under vacuum.

### 2.3. Characterization Techniques

Powder X-ray diffraction (XRD) was used for identifying the nanolayered structures through Rigaku RINT 2200 (Tokyo, Japan) with CuK_ (filtered) as a source of radiation at wavelength λ = 0.154 nm between the angles 2Ѳ = 1.8–50°. Imaging the prepared samples at the nanoscale was performed by JEOL JSM-6330F (Tokyo, Japan) scanning electron microscopy. Energy dispersive X-ray spectroscopy (EDX; JEOL Company, Tokyo, Japan) was used to identify the different elements in the prepared materials through an electron probe micro-analyzer JED 2300. Using a heating rate of 10 °C/min, the thermal analyses of prepared samples were studied up to 800 °C through thermal gravimetric analysis (TGA), differential thermal analysis (DTA), and differential thermal gravimetric (DTG) using a Seiko SSC 5200 apparatus (Tokyo, Japan). The functional groups of the prepared samples were detected by Fourier Transform Infrared (FT-IR) spectroscopy in a wide range of wavenumbers from 425 to 4000 cm^−1^ by Horiba FT-720 (Tokyo, Japan). The optical parameters of the prepared nanomaterials were determined by the diffuse reflectance technique of UV Shimadzu 3600. The reflectance was transformed to absorbance using the software of the spectrophotometer (Shimadzu, Columbia, MD, USA). To measure the solid materials, the spectrophotometer was attached with an ISR-603 (integrating sphere attachment, Shimadzu, Columbia, MD, USA). For measuring liquid samples, the absorption parameters were measured by the conventional UV/VIS Shimadzu 3600.

### 2.4. Photocatalytic Measurements

Photocatalytic degradation of the colored pollutants was studied through determining the photocatalytic activity of the nanolayered structures and its derivatives. The photochemical reactors limited company (Camberley, Surrey, UK) was the source for the quartz immersion well reactor (RQ400), which was used for performing photocatalytic reactions. In this reactor, the immersion lamp was very effective for photochemical reactions because of the direct contact between the solution and the lamp. The double-walls of the immersion well, which were composed of quartz, were used for cooling the lamp during the photocatalytic reactions. The mercury lamp (Camberley, Surrey, UK) 3040/PX0686, which had 400 W medium pressure, was combined with a 400 mL standard reaction flask Model 3308 to carry out the photocatalytic reactions. The major part of the radiation for a medium pressure lamp focuses on the range from 365 to 366 nm. Moreover, it produces small amounts in the ultraviolet region at 254, 265, 270, 289, 297, 302, 313, and 334 nm. In addition, significant amounts of radiation are produced in the visible region at 405–408, 436, 546, and 577–579 nm.

In the current study, the photocatalytic reactions were performed for Acid Green 1, which was used as one of the industrial pollutants. A low concentration of an aqueous solution of the green dye was prepared to be 4 × 10^−4^ M. By measuring the maximum band in the spectrum of the dye, the concentration of the dye can be determined by the law of Beer–Lambert. The characteristic band of Acid Green 1 is at 714 nm. Therefore, the concentration change in the green color was monitored by measuring the absorbance at 714 nm. The mixture of the aqueous solution of the green dye and one of the prepared materials was irradiated with UV light inside the photocatalytic reactor at room temperature. The experiment was performed in the dark for 60 min to avoid the adsorption process between the photocatalyst and the dyes. Moreover, this experiment was carried out without a photocatalyst in the presence of UV light for 60 min to test the stability of the dye. After irradiation with UV light, small doses of the solution were withdrawn at different intervals of time. Using a UV-Vis spectrophotometer, the concentration of the remaining dye in the solution was measured.

## 3. Results

### 3.1. Characterization of the Nanolayered Structures and the Nanohybrids

**Figure 1:**
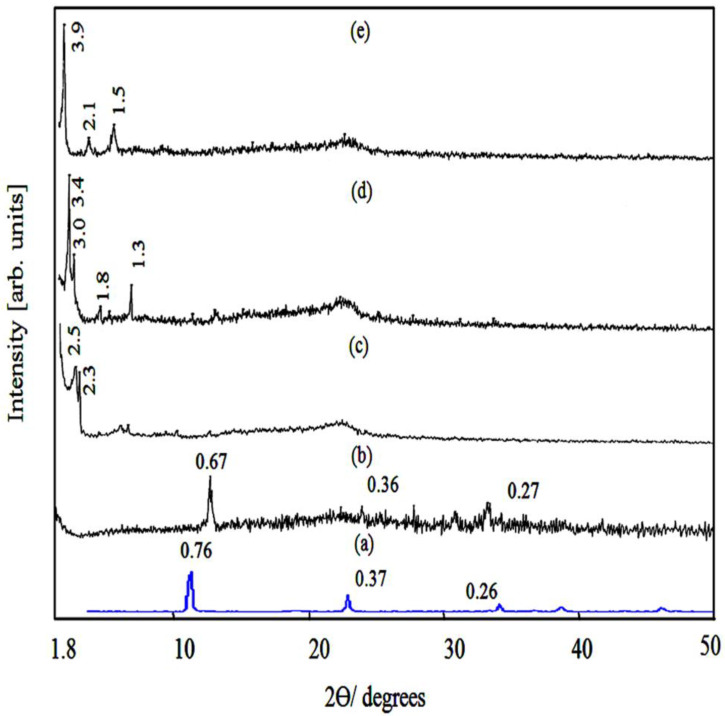
X-ray diffraction patterns of: (**a**) Zn/Al LDH, (**b**) Co/Zn/Ti LDH, (**c**) Co/Zn/Ti-CH_3_(CH_2_)_8_COOH nanohybrid, (**d**) Co/Zn/Ti-(CH_3_(CH_2_)_12_COOH) nanohybrid, and (**e**) Co/Zn/Ti-(CH_3_(CH_2_)_16_COOH) nanohybrid.

To study and characterize the nanolayered structures of Co/Zn/Ti LDH, one of the most familiar nanolayered structures Zn/Al LDH was prepared and characterized for comparison. Figure 1a exhibits the characteristic peaks of the Zn-Al LDH at 2Ѳ = 11.6°, 24.1°, and 34.1° agreeing with the d-spacing at 0.76 nm, 0.37 nm, and 0.26 nm and matching with the JCPDS file No. 37-629.

The highly ordered nanolayers are observed along the axis “c” because of the matching between the successive diffractions planes; d(003) = 2 × d(006) = 3 × d(009). From the interlayered spacing of plane (003), the value c was assessed to be 2.28 nm (3 × 0.76 nm). The value “c” is identical to that reported for the conventional layered double hydroxides and the natural hydrotalcite; 2.28 nm [3]. The X-ray diffraction pattern of the nanolayered structure of Co/Zn/Ti LDH is displayed in Figure 1b. The main peak of Co/Zn/Ti LDH is observed at a d-spacing 0.67 nm. In comparison with the main peak of the usual Zn/Al LDH, this interlayered spacing is smaller. The previous studies of Constantino et al. [41], Yun et al. [42], and Saber et al. [43] indicated that interlayer spacing, 0.76 nm, of the LDH consisting of di- and trivalent cations, decreased to 0.67 nm by removing the interlayered water after drying at 150 °C. This means that the interlayered spacing of LDH can be reduced from 0.76 nm to be 0.67 nm in special conditions. Therefore, the small d-spacing of Co/Zn/Ti LDH can be explained through the presence of tetravalent titanium inside the nanolayers, which created higher positive charges (+2). The attraction forces between these positive charges and the interlayered anions became stronger and led to narrowing the interlayered spacing among the nanolayers from 0.76 nm to 0.67 nm. In comparison with the other peaks of the usual Zn/Al LDH, the X-ray diffraction of Co/Zn/Ti LDH showed the basal peaks of planes (003), (006), and (009) indicating nanolayered structure.

In order to confirm the nanolayered structure of Co/Zn/Ti LDH, the anion exchange property, which is considered one of the most famous properties of LDHs, was investigated through intercalation reactions. A series of long chains of fatty aliphatic acids, monocarboxylic and dicarboxylic, was used for building organic–inorganic nanohybrids based on host–guest interactions.

By using n-capric acid, which has ten carbon atoms, the first nanohybrid was prepared, as shown in Figure 1c. Figure 1c shows that the Co/Zn/Ti-C10 nanohybrid was prepared and the nanolayered structure became clearer. Moreover, the X-ray diffraction pattern showed that the prepared material became more crystalline through observing sharp peaks at low 2Ѳ. The interlayered spacing increased from 0.67 nm to become 2.5 nm and 2.3 nm, indicating two interlayered spacings. In the case of intercalating myristic acid inside Co/Zn/Ti LDH, which has fourteen carbon (CH_3_(CH_2_)_12_COOH), the second nanohybrid Co/Zn/Ti-C14 was produced, as shown in Figure 1d. X-ray diffraction showed more expansion for the d-spacing of the Co/Zn/Ti-C14 nanohybrid, where the main peaks were sharp and were observed at low 2Ѳ agreeing with d-spacing 3.4 nm and 3.0 nm. Moreover, X-ray diffraction revealed a clear nanolayered structure because of the arrangement of the peaks 3.4 nm ≈ 2 × 1.8 nm ≈ 3 × 1.3 nm. The third nanohybrid Co/Zn/Ti-C18 was prepared depending on the intercalation reaction of the long-chain fatty acid stearic acid (CH_3_(CH_2_)_16_COOH) with the Co/Zn/Ti LDH. The X-ray diffraction pattern of Co/Zn/Ti-C18 showed a sharp peak at 2Ѳ = 2.26°, agreeing with the d-spacing 3.9 nm. This means that the interlayered spacing of Co/Zn/Ti-C18 expanded and widened by the longer chain of the aliphatic acid. Moreover, the nanolayered structure was observed through the arrangement of the peaks 3.9 nm ≈ 2 × 2.1 nm ≈ 3 × 1.5 nm.

**Figure 2:**
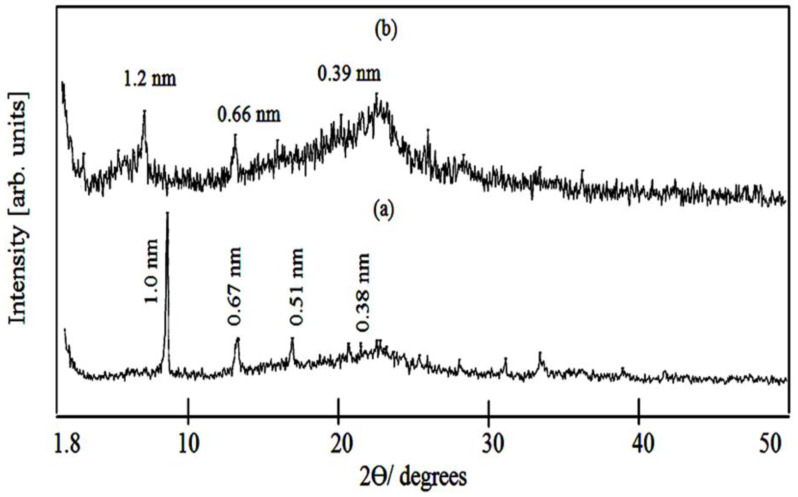
X-ray diffraction patterns of: (**a**) Co/Zn/Ti-(COOH(CH_2_)_6_COOH) nanohybrids and (**b**) Co/Zn/Ti-(COOH(CH_2_)_8_COOH) nanohybrids.

Another kind of nanohybrid was prepared depending on the long chains of dicarboxylic acids, as shown in Figure 2. Suberic acid (COOH(CH_2_)_6_COOH) and sebacic acid (COOH(CH_2_)_8_COOH) were used for intercalating with Co/Zn/Ti LDH to form a limited expansion for the interlayered spacing of the LDH because each molecule of the dicarboxylic acid has two negative charges at its end, and attracts with two layers at the same time. In the case of intercalating suberic acid, one sharp peak is observed at 2Ѳ = 8.8°, agreeing with d-spacing 1.0 nm, as seen in the X-ray diffraction pattern in Figure 2a. In addition, the original peak of the LDH did not disappear, indicating that there are two nanolayered structures: Co/Zn/Ti LDH and Co/Zn/Ti-CC8 nanohybrid. By using sebacic acid for intercalation reaction with the Co/Zn/Ti LDH, the sharp peak shifted to a lower angle 2Ѳ = 7.35°, indicating more widening for the d-spacing of the nanohybrid Co/Zn/Ti-CC10, as shown in Figure 2b. The success of the intercalation reactions with Co/Zn/Ti LDH confirms the formation of the nanolayered structures of Co/Zn/Ti LDH.

**Figure 3:**
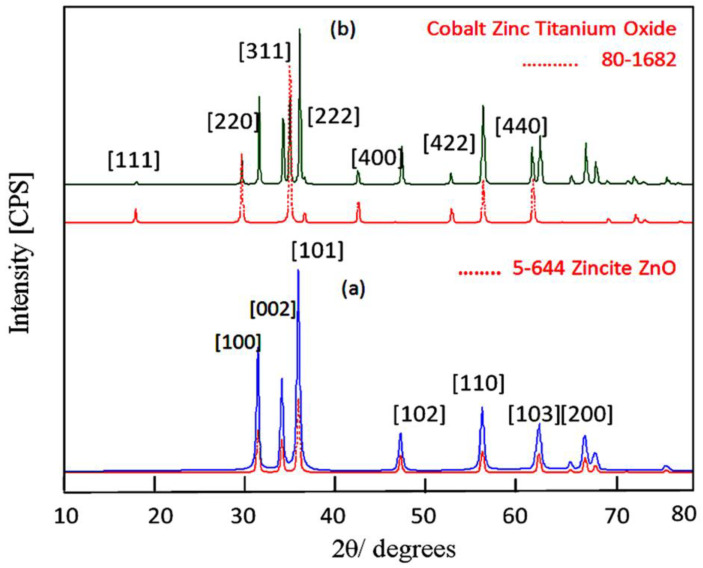
X-ray diffraction patterns of Co/Zn/Ti LDH after calcination at: (**a**) 500 °C and (**b**) 900 °C.

Using thermal treatment for the prepared Co/Zn/Ti LDH at 500 °C, the main peaks of the nanolayered structure were disappeared, as shown in Figure 3a. Moreover, the X-ray diffraction pattern of Co/Zn/Ti-500 showed new peaks at higher angles 2Ѳ = 31.8°, 34.6°, 35.2°, 46.8°, 56.61°, 62.3 °, and 68.18°, agreeing with the reflections of the planes [100], [002], [101], [102], [110], [103], and [200]. These peaks indicate production of a new nanocomposite based on a zinc oxide structure, because these diffraction peaks are matching, and fitting with the standard entire diffraction pattern of the zincite phase of zinc oxides (JCPDS No. 75-576) and (JCPDS No. 5-664). No peaks were observed for titanium or cobalt in the XRD diagram of Co/Zn/Ti-500. This means that Ti and Co are well dispersed and homogeneously distributed inside the structure of the zinc oxide. This finding was confirmed by calcining the Co/Zn/Ti LDH at 900 °C, as shown in Figure 3b. X-ray diffraction of Co/Zn/Ti-900 showed new peaks at 2Ѳ = 18.27°, 29.92°, 35.33°, 36.88°, 42.1°, 53.11°, and 62.36° in addition to the original peaks of zinc oxide. These peaks agree with the reflections of the planes [111], [220], [311], [222], [400], [422], and [440] of cobalt–zinc–titanium oxide according to JCPDS (No. 80-1682). In comparison with the standard diffraction pattern of JCPDS (No. 80-1682), the titanium and cobalt atoms were homogeneously dispersed inside the zinc oxide structure to produce a new phase (Co_0.2_Zn_0.8_)(CoTi)O_4_ after calcining at 900 °C in addition to the original phase of the zinc oxide.

**Figure 4:**
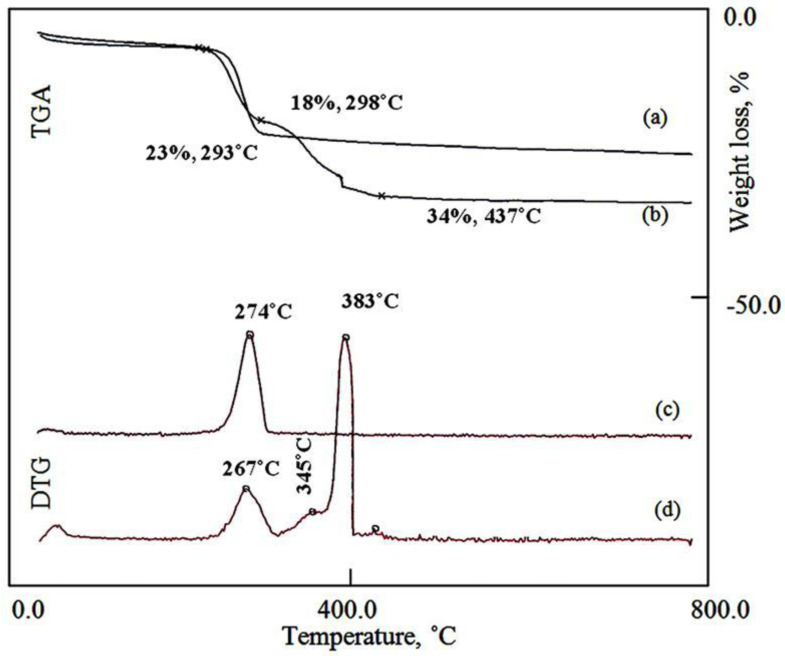
Thermal analyses (TGA and DTG) of: (**a**,**c**) Co/Zn/Ti LDH and (**b**,**d**) Co/Zn/Ti-C10 nanohybrid.

Thermal analyses were used to confirm the formation of Co/Zn/Ti LDH and its nanohybrids. Figure 4 shows the diagrams of thermal thermogravimetric analysis (TGA) and differential thermal thermogravimetric (DTG) of both Co/Zn/Ti LDH and Co/Zn/Ti-C10 nanohybrid. TG diagram revealed that 26% of Co/Zn/Ti LDH was lost at 794 °C, agreeing with the usual behavior of LDH. This weight loss happened through three stages. In the first step, the loss was 3.5% at 220 °C, indicating removal of the surface and interlayered water. The second step has a main weight loss of 14.5%, which was observed in the temperature range from 220 to 298 °C, and represents the decomposition of the interlayered anions of the LDH. The dehydroxylation process of the nanolayers of Co/Zn/Ti LDH occurred in the third step, causing a loss of 3%, and completed at 794 °C. The DTG diagram confirmed the main weight loss of the LDH by a broad peak at 274 °C.

The formation of organic–inorganic nanohybrid Co/Zn/Ti-C10 was approved by the comparison between the TG diagrams of the LDH before and after the intercalation reactions with the organic species as shown in Figure 4a,b. The intercalation reaction caused a large alteration for the main weight loss of the Co/Zn/Ti-C10 nanohybrid in addition to a big shift for the decomposition temperature of the interlayered anions, where the main weight loss of the nanohybrid was observed in the temperature range from 220 to 437 °C and reached 34%. This means that the interlayered carbonate anions of the LDH changed to be organic anions. Moreover, the DTG diagram of Co/Zn/Ti-C10 nanohybrid showed more than three peaks at 267 °C, 345 °C, and 383 °C for the decomposition of the interlayered anions of the nanohybrid, confirming the results of the TG curve, as seen in Figure 4d.

**Figure 5:**
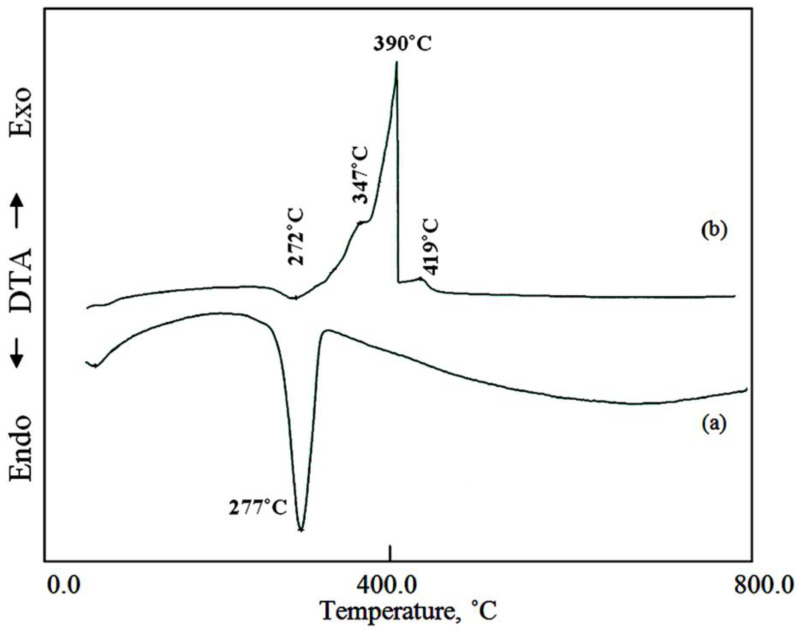
Thermal analyses (DTA) of: (**a**) Co/Zn/Ti LDH and (**b**) Co/Zn/Ti-C10 nanohybrid.

Another tool of the thermal analysis was used to identify the different kinds of reactions, which happened inside the nanolayered structure during the increase in temperature. Differential thermal analysis (DTA) was measured for the Co/Zn/Ti LDH and Co/Zn/Ti-C10 nanohybrids, as shown in Figure 5. In the case of the Co/Zn/Ti LDH, only one endothermic peak was observed at 277 °C, agreeing with the decomposition reaction of the interlayered carbonate anions. Meanwhile, for the nanohybrid, Figure 5b showed four exothermic peaks at 272 °C, 347 °C, 390 °C, and 419 °C. These exothermic peaks indicate a release of a large amount of heat during the decomposition of the interlayered anions because of the oxidation reactions of the hydrocarbon structure of the interlayered organic products. The DTA data confirmed the production of an organic–inorganic nanohybrid during the intercalation reactions of the Co/Zn/Ti LDH. The thermal analyses data agree with the XRD results for confirming the formation of nanolayered structures and nanohybrids of Co/Zn/Ti.

The structure of both Co/Zn/Ti LDH and its nanohybrid were confirmed by the FT-IR technique through identifying their function groups. The FT-IR data were summarized in Table 1. For Co/Zn/Ti LDH, the stretching mode of the hydroxyl groups was observed at 3392 cm^−1^, agreeing with the reported results for the conventional LDHs [1,3]. The bending mode band of water molecules was observed at 1623 cm^−1^. The bands at 1504 cm^−1^ and 1394 cm^−1^ are due to mode ν_3_ of the interlayer carbonate anions [3,5]. The bands of metal oxides are recorded at wavenumbers less than 500 cm^−1^, as seen in Table 1. These results indicate that the prepared Co/Zn/Ti LDH has a layered double-hydroxide structure, confirming the presence of carbonate anions and the small amount of water inside the interlayer space.

In the case of Co/Zn/Ti-C10 nanohybrids, the presence of organic compounds was confirmed by the results of FT-IR data, as shown in Table 1. The carbon–hydrogen stretch absorption bands were observed at 2956–2850 cm^−1^ [44,45,46]. Moreover, the carbon–hydrogen bending band was observed at 1465 cm^−1^. In addition, Table 1 shows two peaks at 1540 cm^−1^ and 1396 cm^−1^. The absorption at 1540 cm^−1^ is assigned to the symmetric stretching vibration of carboxylate, and the absorption at 1396 cm^−1^ is due to the asymmetric stretching vibration of carboxylate [44,45,46]. In addition, the absorption band of the hydroxyl group did not disappear while the absorption bands of carbonate anions disappeared. This means that Co/Zn/Ti-C10 nanohybrids were successfully formed after intercalation reactions between n-capric acid and Co/Zn/Ti LDHs.

**Figure 6:**
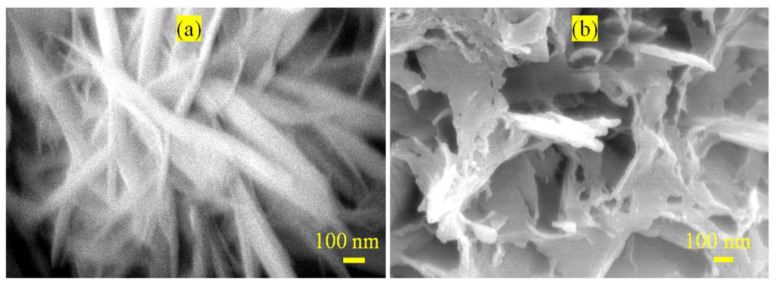
SEM images of: (**a**) Co/Zn/Ti LDH and (**b**) Co/Zn/Ti-C10 nanohybrid.

The nanolayered structures of the conventional layered double hydroxides show plate-like morphology with hexagonal geometry [3,5]. The morphology of the prepared nanostructures was detected through imaging Co/Zn/Ti LDH by scanning electron microscopy after coating its surface with a platinum thin film to increase the resolution of their images, as seen in Figure 6. Figure 6a shows nanoplatelets looking like fibers with irregular shapes. Moreover, it indicates that Co/Zn/Ti LDH consists of aggregates of fibers with diameters of 100 nm. Through intercalation reactions, Figure 6b reveals that the morphology changed to become a network of platelets at the nanoscale.

**Figure 7:**
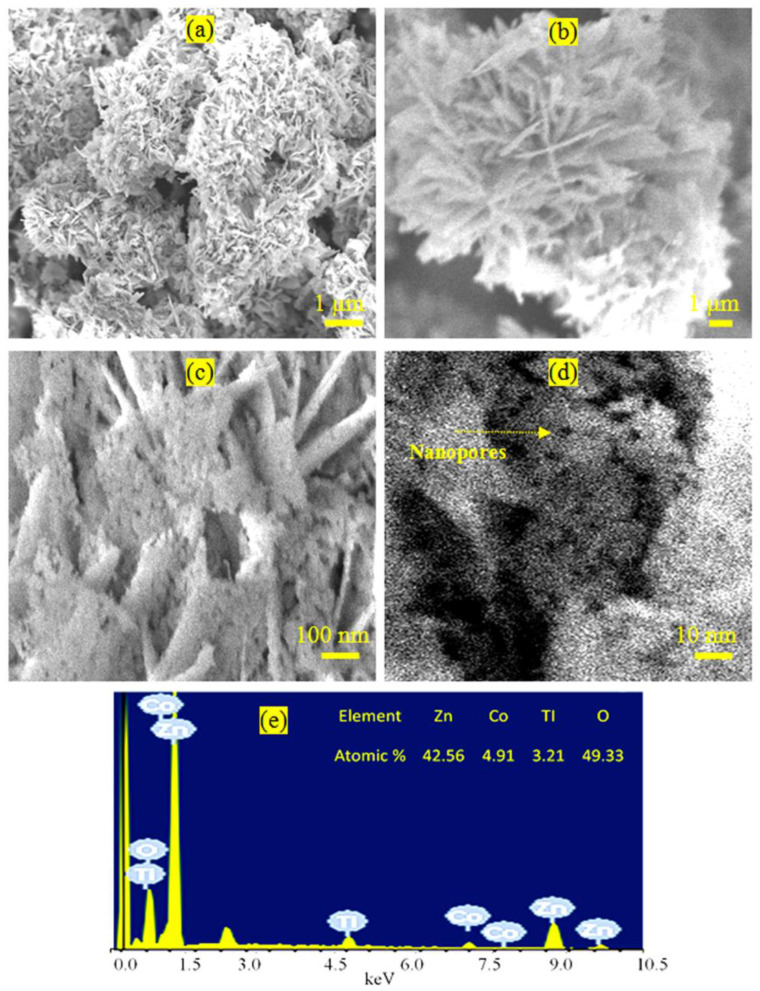
SEM images of Co/Zn/Ti-500: (**a**) the first spot, (**b**) the second spot, (**c**) after magnification 100 nm, (**d**) after magnification 10 nm and (**e**) EDX spectrum.

Similar morphology was observed for Co/Zn/Ti LDH after calcination at 500 °C, as shown in Figure 7. The SEM images of Co/Zn/Ti-500 showed nanoplates, as seen in Figure 7a. Figure 7b reveals that these nanoplates aggregated with special orientations to produce flower-shapes. Through magnification, Figure 7c indicates that the nanoplates have nanopores. These nanopores are clear in Figure 7d, and marked by arrows. These nanopores were formed during the evaporation of the interlayered water and released the volatile gases which produced from the thermal decomposition of the interlayered anions of the layered structure of Co/Zn/Ti LDH.

Energy-dispersive X-ray spectrometry (EDX) analysis has provided clear information on the different elements in the outermost layers of Co/Zn/Ti-500. The EDX spectrum confirmed that the main composition of Co/Zn/Ti-500 is zinc oxide because the percentages of zinc and oxygen are 42.56% and 49.33%, as seen in Figure 7e. In addition, cobalt and titanium are detected in the spectrum of Co/Zn/Ti-500 by 4.91% and 3.21%; respectively. The EDX results agree with the XRD results, confirming that the sample Co/Zn/Ti-500 has a ZnO structure doped with titanium and cobalt.

**Figure 8:**
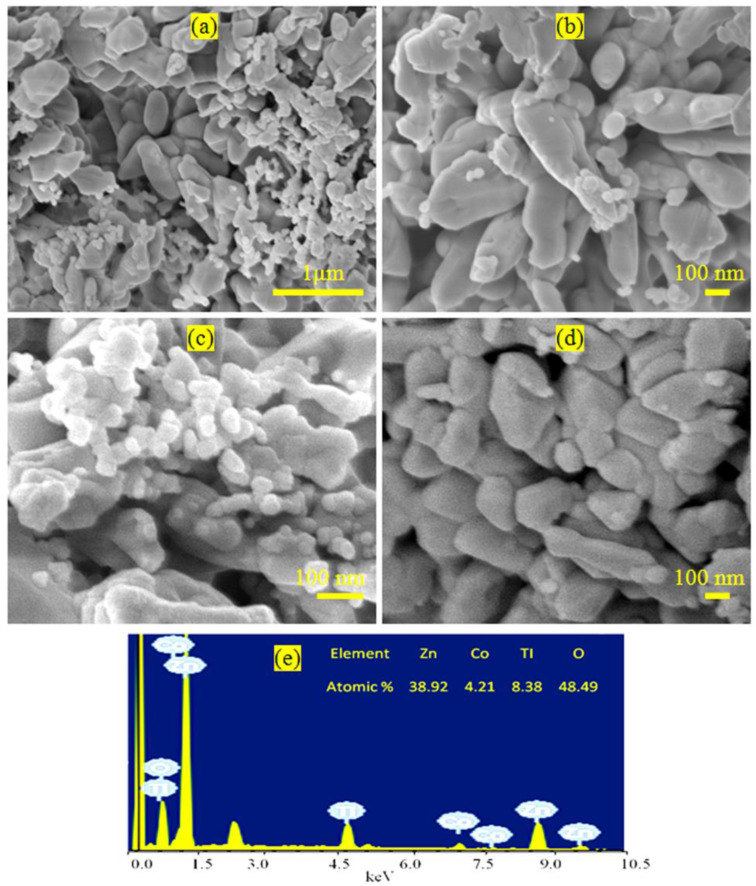
SEM images of Co/Zn/Ti-900: (**a**) the first spot, (**b**) the second spot, (**c**) the third spot, (**d**) the fourth spot, and (**e**) EDX spectrum.

After thermal treatment of Co/Zn/Ti LDH at 900 °C, the SEM images showed two kinds of particles: nanoparticles and microparticles, as seen in Figure 8. Agreeing with the results of X-ray diffraction, there are two phases. Figure 8b shows that the nanoplatelets, which were produced from calcination at 500 °C, started to aggregate and combine together after thermally treating them at 900 °C to produce a cobalt–zinc–titanium oxide nanocomposite. This means that the microparticles are due to the cobalt–zinc–titanium oxide nanocomposite. At the same time, the nanoplates and nanopores, which were formed at 500 °C, collapsed through the sintering process at the high temperature, as shown in Figure 8c. The formation of the multidoped structure of the zinc oxide was confirmed by EDX analysis, as shown in Figure 8e, where a high percentage of zinc 38.92% was detected in the spectrum of Co/Zn/Ti-900. In addition, cobalt and titanium were observed with low percentages in the spectrum of EDX in Figure 8e.

### 3.2. Optical Properties

The important information of the optical properties of the nanolayered structures of Co/Zn/Ti LDH, before and after calcination, could be provided by UV–Vis absorption spectroscopy.

**Figure 9:**
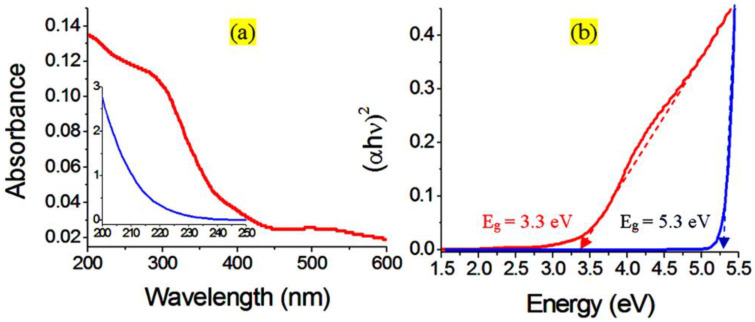
The prepared Co/Zn/Ti LDH (inset, the usual Zn/Al LDH): (**a**) Absorbance spectrum and (**b**) Band gap energy (the blue color means Zn/Al LDH and the red color means Co/Zn/Ti LDH).

Figure 9a displays the UV-Visible absorbance spectrum of the Co/Zn/Ti LDH. Three peaks are observed at 500 nm, 300 nm, and 200 nm. In comparison with the usual Zn/Al LDH, which is shown in Figure 9a (inset), a clear shift toward the visible region was observed for the Co/Zn/Ti LDH. Moreover, the absorbance edge was shifted to become near 600 nm in the case of the Co/Zn/Ti LDH. This means that the insertion of both cobalt and titanium inside the nanolayers of the LDH improved the optical properties of the nanolayered structure of the LDH.

By calculating the band gap energy depending on the scientific formula between the energy of the incident photon (hν) and the absorbance coefficient of the Co/Zn/Ti LDH, the following equation [27] was used:

(αhν)^2^ = constant (hν − E_g_)

In this equation, the Planck’s constant (h) is used with the speed and the wavelength of light to express the energy (E). The value (α) represents the coefficient of the absorbance of the Co/Zn/Ti LDH. The allowed direct transition from the valence band to the conduction band could be expressed by the exponent (2). If the value (αhν)^2^ equals zero, the incident photon energy will be equaled over the energy band gap. In this way, the band gap energy of the prepared Co/Zn/Ti LDH could be measured through plotting the energy (hν) in *X*-axis and (αhν)^2^ in *Y*-axis. The extension of the straight line toward the *X*-axis led to determining the band gap energy.

**Figure 10:**
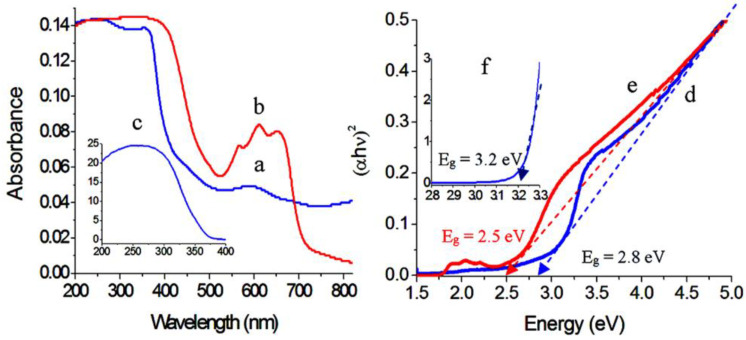
Absorbance spectra of (a) Co/Zn/Ti-500, (b) Co/Zn/Ti -900, (c) Zn/Al-500 and the band gap energy of (d) Co/Zn/Ti-500, (e) Co/Zn/Ti -900, (f) Zn/Al-500.

Figure 9b displays the band gap energy of the Co/Zn/Ti LDH and the usual Zn/Al LDH. The band gap energy of Co/Zn/Ti LDH was observed at 3.3 eV. For the usual Zn/Al LDH, the band gap energy was 5.3 eV. In comparison with the usual LDH, the energy band gap of the Co/Zn/Ti LDH shifted from 5.3 eV toward the lower value 3.3 eV. This shift confirms that the presence of cobalt and titanium inside the structure of LDHs improved its optical properties. This improvement extended to the oxide’s form, which was produced form these nanolayered structures. Figure 10a,b display the UV-Visible absorbance spectra of the Co/Zn/Ti LDH after calcination at 500 °C and 900 °C. They show three maxima at 600 nm, 400 nm, and 250 nm for the sample Co/Zn/Ti-500. In the case of the sample Co/Zn/Ti-900, four maxima were observed at 400 nm, 600 nm, 650 nm, and 700 nm. For Zn/Al-500, Figure 10c shows only one maximum at 250 nm. The comparison between Co/Zn/Ti-500, Co/Zn/Ti-900, and Zn/Al-500 showed a strong effect in the presence of cobalt and titanium inside the structure of zinc oxide for improving their optical absorbance.

This strong effect indicates an enhancement for the optical properties of the Co-Ti-dual-doped zinc oxide which was produced from Co/Zn/Ti LDHs. This enhancement was confirmed by calculating the energy of the band gap. Figure 10d,e indicate that the Co-Ti-dual-doped zinc oxide has a lower band gap energy than the zinc oxide based on the usual LDH and the pure zinc oxide, where the energies of the band gap of Co/Zn/Ti-500 and Co/Zn/Ti-900 are 2.8 eV and 2.5 eV, respectively. For Zn/Al-500, Figure 10f shows that the Al-doped ZnO nanoparticles have a band gap energy at 3.2 eV. In comparison with the Al-doped ZnO nanoparticles, the Co-Ti-dual-doped ZnO caused narrowing for the band gap from 3.2 eV to 2.5–2.8 eV. This narrowing for the band gap energy and shifting for the absorbance of the Co/Ti-dual-doped zinc oxide toward a higher wavelength indicate that the combination of cobalt and titanium with zinc oxide through the construction of nanolayered structures improved the optical parameters of the zinc oxide to become more active in optical applications.

### 3.3. Photocatalytic Decomposition of the Green Pollutants

**Figure 11:**
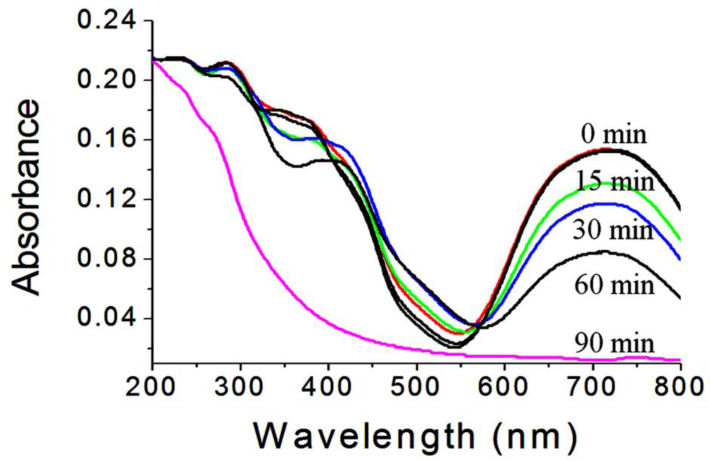
Absorbance spectra of AG1 with different irradiation times in UV light in the presence of the nanolayered structure of Co/Zn/Ti LDH.

**Figure 12:**
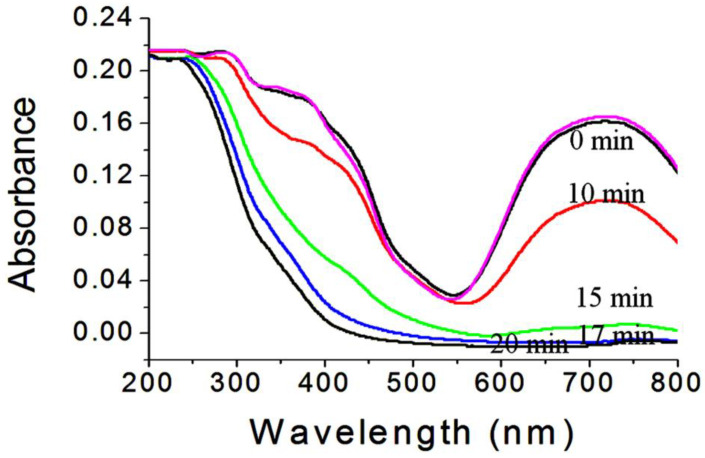
Absorbance spectra of AG1 with different irradiation times in UV light in the presence of Co/Zn/Ti-500.

**Figure 13:**
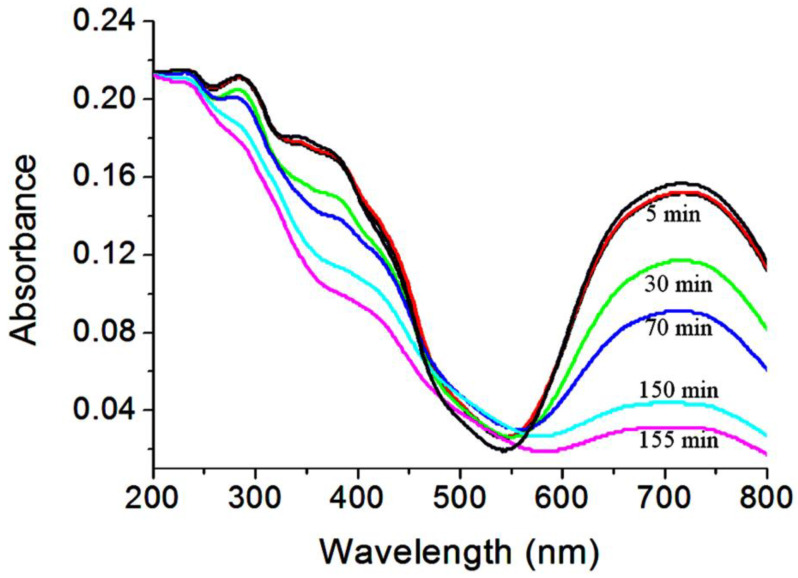
Absorbance spectra of AG1 with different irradiation times in UV light in the presence of Co/Zn/Ti-900.

The fast and effective removal of industrial pollutants is one of the main objectives of the current study. Therefore, photocatalytic activities of the nanolayered structure of Co/Zn/Ti LDH and its nanocomposites were tested and compared with the most familiar photocatalysts in the field of the degradation of green dyes. Moreover, the Al-doped ZnO and the pure zinc oxide were used as photocatalysts for comparison. In this direction, the concentration of the dye of Acid Green 1 was determined during the radiation of UV light in the presence of one of the prepared materials by measuring the absorbance of the green color. The drop in the absorbance of the green color at the maximum band at 714 nm indicates the decomposition of the principal structures of the dyes, while the decomposition of the internal structure of the dyes could be followed from the absorption bands at 320 nm, 283 nm and 232 nm, as seen in Figure 11, Figure 12 and Figure 13.

The green dye was stable toward the UV irradiation because the blank experiment, which was performed without photocatalysts for 60 min, showed no change for the concentration, and was considered 0 min in the study, as seen in Figure 11, Figure 12 and Figure 13. The photocatalytic decomposition of the green dye was studied as a function of the radiation time of UV light in the presence of the photocatalysts. When the used sample was stirred with the green solution of dye for 60 min in the dark, no change was detected for the concentration of the dye. During the radiation of the aqueous solution of the green dye with UV light, the photocatalytic decomposition of AG1 was observed. The obtained results are illustrated in Figure 11, Figure 12 and Figure 13.

Figure 11 shows that the photocatalytic decomposition of AG1 under UV radiation by the nanolayered structure of Co/Zn/Ti LDH increases with the rise in radiation time. At 90 min of radiation time, complete decolorization and decomposition of the dyes were attained using Co/Zn/Ti LDH. In the case of using the nanolayered structure after calcination at 500 °C, the green color was completely disappeared after 17 min indicating a complete mineralization of the dye. Moreover, Figure 12 showed that the maximum band at 714 nm gradually decreased to attain 0 after 17 min. At the same time, the other absorption bands at 320 nm, 283 nm, and 232 nm disappeared after 17 min. This means that the dual Co-Ti-doped zinc oxide Co/Zn/Ti-500 is very effective for removing the green dyes from water in the presence of UV light. After calcination at 900 °C, the activity of the photocatalyst Co/Zn/Ti-900 became low, because the degradation of the green dye did not complete, although the time of UV radiation was 155 min, as shown in Figure 13. This high drop in the activity of Co/Zn/Ti-900 could be explained according to the SEM images. The results of SEM indicated that the sample Co/Zn/Ti-900 lost its nanostructure and converted to the microscale through collapsing the 3D porous structure of the dual Co-Ti-doped zinc oxide because of the high temperature (900 °C). The high temperature caused the sintering process for the photocatalyst, leading to blockage of the optical active centers.

Through comparison with the Al-doped zinc oxide, the high activity of the dual Co-Ti-doped zinc oxide Co/Zn/Ti-500 is very clear, because the complete decolorization and degradation of the dyes in the presence of the Al-doped zinc oxide happened after 160 min. For the pure zinc oxide, the degradation for the green dye reached 100% after 51 min of UV-radiation. In comparison with the optical activity of the pure zinc oxide, the complete decolorization and degradation of the dyes was more than two times faster for the dual Co-Ti-doped zinc oxide Co/Zn/Ti-500.

## 4. Discussion

The use of the direct UV light on the prepared dual-doped zinc oxide with cobalt and titanium was very effective for the complete decolorization and mineralization of the dye after 17 min of UV-irradiation. Figure 12 showed that the main absorbance band of the green dye at 714 nm reached 0 after 17 min, indicating the degradation of the main hydrocarbon chains of the green dye. Moreover, the bands at 320 nm, 283 nm, and 232 nm, which belong to the intermediate organic phenyl groups, disappeared, confirming the complete degradation of the green dye.

Through comparison with the results of Baliarsingh et al. [47,48] and Wang et al. [49], the prepared and calcined Co/Zn/Ti LDH is optimum. Baliarsingh et al. investigated the effect of different kinds of LDHs such Co-Cr LDH, Ni-Cr LDH, Cu-Cr LDH, and Zn-Cr LDH on the photocatalytic degradation of methyl orange dye (MO). Among these LDHs, Co-Cr LDH showed the highest photo-activity at 90% MO removal after 3 h of light radiation. In the the study by Wang et al., the removal of MO by Ni-Al LDH composite films reached 55.30% after 60 min of light radiation. After 120 min of light radiation, the removal of MO attained 79.16%.

**Scheme 1:**
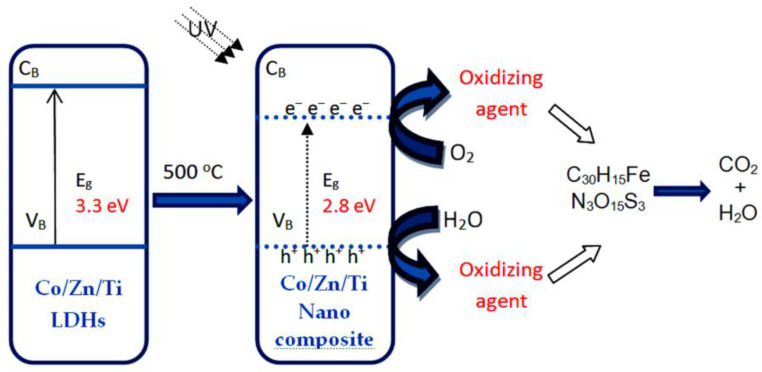
The band gap energy of Co/Zn/Ti LDHs and its nanocomposite and the mechanism of photocatalytic degradation of AG1 dyes.

The theoretical basis [1,3,20,21,22,23,24,25,26,27,28,29,30] for the mechnism of photocatalysts depends on the separation processes between electrons and holes when exposed to UV light, as seen in Scheme 1. Due to the photons of the UV light, the electrons are excited in the valence band and jump to the conduction band, leaving holes in the valence band, as shown in Equation (1). Accordingly, the oxygen molecules are transformed through the excited electrons to become the strong oxidizing agent ^•^O_2_¯. Moreover, the water molecules are reacted with the holes to produce the highly oxidizing ^•^OH radicals, as shown in the following equations:Co-Ti-Zn-O 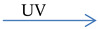 h^+^ (Valence band) + e^−^ (Conduction band)(1)
O_2_ + e^−^

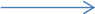
 •O_2_^−^(2)
H_2_O + h^+ 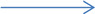 •^OH + H^+^
(3)

In the current study, the high photo catalytic activity of the dual Co/Ti-doped zinc oxides Co/Zn/Ti-500, which were produced from the calcination of Co/Zn/Ti LDH at 500 °C, could be explained by three factors. The first factor depends on the small nanosizes of Co/Zn/Ti-500, which can create new photo-active centers. The second factor focuses on the narrowing of the band gap energy from 3.2 eV to 2.8 eV, which accelerates the separation process between the electrons and holes. The third one is due to the porous structure of Co/Zn/Ti-500 that could make trapping and confinement for pollutants. These three factors were combined to increase the photo catalytic activity of Co/Zn/Ti-500 through producing a large amount of strong oxidizing agents, as shown in Equations (2) and (3). These oxidizing agents could convert the hydrocarbons to carbon dioxide and water, as seen in Scheme 1.

The low optical activity of Co/Zn/Ti-900 could be explained through SEM images. The results of SEM indicated that the sample Co/Zn/Ti-900 lost its nanostructure and converted to the microscale because the high temperature (900 °C) caused sintering for the photocatalyst, leading to the blockage of the optical active centers. This means that two of the three factors became inactive. The sample Co/Zn/Ti-900 lost its nanosize and optical active centers. In addition, the trapping and confinement processes of pollutants could not happen because of the disappearance of the porous structure, as shown in the SEM images.

Although the nanolayered structure of Co-Ti-Zn LDH can make trapping and confinement for the pollutants, the first and second factors were inactive. The low optical activity of Co-Ti-Zn LDH is due to the large band gap energy and the low optical active centers. This means that Co-Ti-Zn LDH needs to capture or absorb photons of energy equal to, or higher than, 3.3 eV to activate separation processes between the electrons and holes.

These explanations were confirmed by studying the kinetic reactions of the photocatalytic degradation of the green dyes for Co-Ti-Zn LDH, Co/Zn/Ti-500, and Co/Zn/Ti-900, according to the following relation:

ln [C_o_]/[C] = kt

whereas [C_o_] is the concentrations of the dye at time t = 0. The value [C] is the concentration of the dye at time (t). The rate constant of the reaction is k.

**Figure 14:**
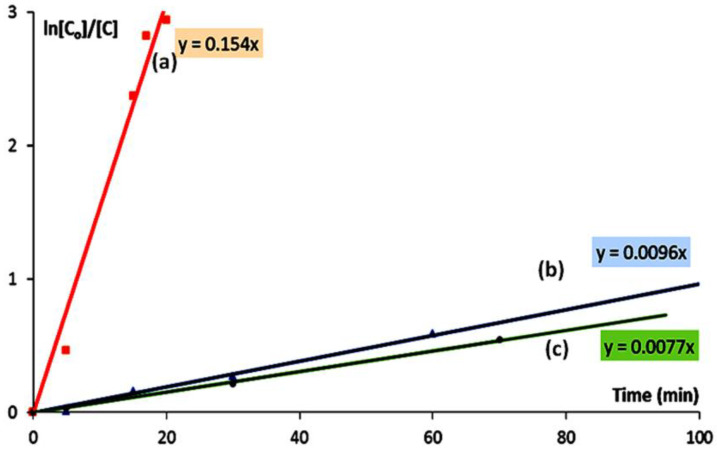
Kinetic studies obtained for the photocatalytic degradation of the green dyes in the UV light using: (**a**) Co/Zn/Ti-500, (**b**) Co-Ti-Zn LDH, and (**c**) Co/Zn/Ti-900.

By plotting ln [C_o_]/[C] on the *Y*-axis with the time (t) on the *X*-axis, the kinetic diagrams of the photocatalytic degradation of the green dyes using the nanolayered structures of Co-Ti-Zn LDH and the dual Co-Ti-doped zinc oxide Co/Zn/Ti-500 and Co/Zn/Ti-900 are displayed in Figure 14.

Figure 14b shows a straight line for the green dye using UV light and employing the nanolayered structures of Co-Ti-Zn LDH as a photocatalyst. It indicates that this photocatalytic degradation process is a pseudo-first-order kinetic reaction with a rate constant of 0.0096 min^−1^. In the case of using the dual-doped zinc oxide Co/Zn/Ti-500, the rate constant increased fifteen times to become 0.154 min^−1^, as seen in Figure 14a. The reduction in the rate constant of the photocatalytic degradation process for Co/Zn/Ti-900 was observed at 0.0077, indicating the blocking of the optical active centers because of the high temperature. For comparison, the rate constant of the same reaction was calculated for the Al-doped zinc oxide based on the usual LDH to be 0.01 min^−1^. The comparison indicated that the dual-doped zinc oxide Co/Zn/Ti-500 caused more than fifteen times faster degradation for the green dyes. The kinetic study confirmed that the high optical activity of the dual-doped zinc oxide Co/Zn/Ti-500 accelerated the degradation of the green dyes.

## 5. Conclusions

In the current research, the preparation of Co/Ti/Zn nanolayered structures was used for achieving many objectives. The first objective was the conversion of nanolayered structures to a series of organic–inorganic nanohybrids based on Co/Ti/Zn LDHs through host–guest interactions for the first time. It was achieved by using long chains of hydrocarbons; monocarboxylic acids and dicarboxylic acids. XRD results, FTIR spectra, and thermal analyses confirmed the formation of five heterostructured nanohybrids with interlayered spacing from 2.3 nm to 3.9 nm. The second aim focused on the improvement of the optical properties of zinc oxide through the building of dual Co/Ti-doped zinc oxide nanocomposites, which were produced after thermal treatment. SEM results and EDX analyses showed conversion of 2D nanolayered structures to 3D porous structures in the dual Co/Ti-doped zinc oxide nanocomposites. The optical properties showed narrowing for the band gap energy of the zinc oxide after doping with cobalt and titanium from 3.3 eV to be 2.8 eV, indicating a highly photo-active nanocomposite. The third objective was achieved for the purification of the polluted water by the dual Co/Ti-doped zinc oxide nanocomposite at 17 min. The kinetic study confirmed the positive effect for accelerating the photocatalytic degradation of the green dyes in the UV-light using the dual Co/Ti-doped zinc oxide nanocomposites. Finally, based on the above results, we can conclude that the ternary system of the Co/Ti/Zn nanolayered structures introduces a new track for designing optical nanocomposites for purifying water. Moreover, it considers a new additive for building hetero-structured nanohybrids.

## Figures and Tables

**Table 1:** FT-IR data of Co/Zn/Ti LDH and Co/Zn/Ti-C10 nanohybrids.

Assignment	Wavenumber (cm^−1^)	
	Co/Zn/Ti LDH	Co/Zn/Ti-C10 Nanohybrids
ν_OH_	3392	3446
Absorption bands (C–H)	-	2956, 2917, 2850, 1465
δ_H2O_	1623	-
Absorption bands of carboxylate (COO−)	-	1540, 1396
Carbonate (ν_3_)	1504, 1394	
M-O	478	480

## Data Availability

Data supporting the reported results will be provided by the authors upon request.
